# Combining Battery‐Type and Pseudocapacitive Charge Storage in Ag/Ti_3_C_2_T*_x_* MXene Electrode for Capturing Chloride Ions with High Capacitance and Fast Ion Transport

**DOI:** 10.1002/advs.202000621

**Published:** 2020-08-27

**Authors:** Mingxing Liang, Lei Wang, Volker Presser, Xiaohu Dai, Fei Yu, Jie Ma

**Affiliations:** ^1^ State Key Laboratory of Pollution Control and Resource Reuse College of Environmental Science and Engineering Tongji University Shanghai 200092 P. R. China; ^2^ College of Marine Ecology and Environment Shanghai Ocean University Shanghai 201306 P. R. China; ^3^ Research Center for Environmental Functional Materials College of Environmental Science and Engineering Tongji University 1239 Siping Road Shanghai 200092 P.R. China; ^4^ Shanghai Institute of Pollution Control and Ecological Security Shanghai 200092 P.R. China; ^5^ INM – Leibniz Institute for New Materials Campus D2 2 Saarbrücken 66123 Germany; ^6^ Department of Materials Science and Engineering Saarland University Campus D2 2 Saarbrücken 66123 Germany

**Keywords:** battery behavior, capacitive deionization, chloride‐ion capturing, pseudocapacitive behavior, Ti_3_C_2_T*_x_*/Ag

## Abstract

The recent advances in chloride‐ion capturing electrodes for capacitive deionization (CDI) are limited by the capacity, rate, and stability of desalination. This work introduces Ti_3_C_2_T*_x_*/Ag synthesized via a facile oxidation‐reduction method and then uses it as an anode for chloride‐ion capture in CDI. Silver nanoparticles are formed successfully and uniformly distributed with the layered‐structure of Ti_3_C_2_T*_x_*. All Ti_3_C_2_T*_x_*/Ag samples are hydrophilic, which is beneficial for water desalination. Ti_3_C_2_T*_x_*/Ag samples with a low charge transfer resistance exhibit both pseudocapacitive and battery behaviors. Herein, the Ti_3_C_2_T*_x_*/Ag electrode with a reaction time of 3 h exhibits excellent desalination performance with a capacity of 135 mg Cl^−^ g^−1^ at 20 mA g^−1^ in a 10 × 10^−3^ m NaCl solution. Furthermore, low energy consumption of 0.42 kWh kg^−1^ Cl^−^ and a desalination rate of 1.5 mg Cl^−^ g^−1^ min^−1^ at 50 mA g^−1^ is achieved. The Ti_3_C_2_T*_x_*/Ag system exhibits fast rate capability, high desalination capacity, low energy consumption, and excellent cyclability, which can be ascribed to the synergistic effect between the battery and pseudocapacitive behaviors of the Ti_3_C_2_T*_x_*/Ag hybrid material. This work provides fundamental insight into the coupling of battery and pseudocapacitive behaviors during Cl^−^ capture for electrochemical desalination.

## Graphical abstract

A Ti_3_C_2_T*_x_*/Ag hybrid is prepared and exhibits exceptional electrochemical desalination rate and capacity, which can be ascribed to the synergistic effect between the battery (Ag nanoparticles) and pseudocapacitive behaviors (layer‐structure Ti_3_C_2_T*_x_*). MXene serves a dual role as an intercalation electrode and facile, electron‐conductive network to capitalize on the Ag/AgCl conversion reaction.

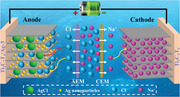

## 1 Introduction

The growing world population, which has been accompanied by a rapid expansion of industry and increased development in agriculture, has led to the need for an increasing amount of fresh water for human beings to make progress.^[^ ^1^ ^]^ Considering the abundance of seawater on Earth, it is important to remove NaCl, which is a great component of brine, to generate fresh water. For instance, chloride ions account for 55% of the total salinity, and the removal of Cl^−^ is a vital task to decrease the total salinity of seawater.^[^ ^2^ ^]^ Currently, thermal evaporation,^[^ ^3^ ^]^ electrodialysis,^[^ ^4^ ^]^ multistage flash distillation,^[^ ^5^ ^]^ reverse osmosis,^[^ ^6^ ^]^ and electrochemical oxidation^[^ ^7^ ^]^ are considered effective techniques for removing NaCl from seawater. However, their considerable energy consumption, pollution, and high cost have limited their large‐scale application.^[^ ^8^ ^]^

Hence, developing facile, feasible, and highly efficient energy utilization technology is beneficial for applications to desalinate seawater. In this context, capacitive deionization (CDI) is widely considered a promising desalination technology, especially for brackish water, due to its high energy efficiency and easy generation compared with those of traditional desalination technology.^[^ ^9^ ^]^ Over the last years, there has been a clear transition from first‐generation CDI by the use of ion electrosorption and nanoporous carbon toward second‐generation CDI based on charge‐transfer materials and processes.^[^ ^10^ ^]^ This has enabled CDI to allow for direct seawater desalination and added additional features, such as ion selectivity. While many Na^+^‐capture electrodes have been investigated, only a few Cl^−^‐capture materials are available in aqueous media, which hinders the further improvement for electrochemical desalination.

In this regard, there are mainly three kinds of desalination mechanisms with Cl^−^ capture: an electrical double layer, pseudocapacitive behavior, and battery behavior. Electrical double‐layer materials primarily consist of carbon‐based electrodes, such as active carbon, porous carbon, and graphene, which have characteristics of low cost, good stability, and cyclability.^[^ ^11^ ^]^ However, it has been shown that the desalination capacity of electrical double‐layer electrodes is limited even though carbon‐based materials with a high specific surface area and good conductivity are used.^[^ ^12^ ^]^ To increase the Cl^−^ removal capacity, Cl^−^ ions should be stored through the formation of chemical bonds rather than by an electric double layer along surfaces or in nanopores.^[^ ^13^ ^]^ Different from carbon materials, faradaic materials store ions via intercalation (pseudocapacitive or battery behavior), or conversion reactions (battery behavior).^[^ ^14^ ^]^ Among the state‐of‐the‐art advances in ionic intercalation electrodes for Cl^−^ capture, MXene (2D transition metal carbides, carbonitrides, and nitrides)^[^ ^15^ ^]^ and polypyrrole chloride have been extensively explored. Srimuk et al. investigated the capability of Mo_1.33_CT*_x_*‐MXene for removing cations and anions by ion intercalation.^[^ ^16^ ^]^ In the MXene community, T*_x_* denotes the presence of surface functionalities created by the synthesis process. Such electrodes display a desalination capacity of 5 mg_NaCl_ g^−1^ in 5 × 10^−3^ m NaCl and 15 mg_NaCl_ g^−1^ in 600 × 10^−3^ m NaCl with a charge efficiency up to 95%. Considering the unfavorable effect of strong van der Waals forces between interlayers on the insertion/release of ions, Bao et al. reported a porous Ti_3_C_2_T*_x_* MXene that was produced through a vacuum freeze‐drying process and was employed to prevent the restacking behavior of MXene nanosheets.^[^ ^14a^ ^]^ This porous Ti_3_C_2_T*_x_* MXene has enhanced specific and volumetric capacitance, an eletrosorption capacity of 45 mg_NaCl_ g^−1^ in a 10 000 mg L^−1^ NaCl solution and good cycling stability (up to 60 cycles). In contrast, polypyrrole chloride has low Coulombic efficiency (64% at the first cycle) and large capacity fading (decreased from 105 to 45 mAh g^−1^ after 15 cycles), although it possesses good electroconductibility and electrochemical reversibility.^[^ ^17^ ^]^

Battery‐behavior electrodes derived from conversion reactions have also attracted vast attention. Gao et al. prepared VOCl for Cl^−^ storage with a reversible capacity of 113 mAh g^−1^ at a specific current of 522 mA g^−1^ even after 100 cycles.^[^ ^18^ ^]^ A nanocrystalline Bi‐foam electrode that provided efficient and high capacity Cl^−^ storage was reported; the above electrode stored Cl^−^ ions in the form of BiOCl.^[^ ^19^ ^]^ Silambarasan and Joseph proposed a charge compensation of redox polymers for Cl^−^ storage.^[^ ^2^ ^]^ ≈164 mg L^−1^ of Cl^−^ ions were removed from natural seawater over a redox‐polysilsesquioxane (redox‐PSQ) film with 98% Coulombic efficiency. However, the immobilization of the redox couple (ferrocyanide) introduced into the cationic PSQ film is achieved merely through electrostatic interactions, which might lead to poor stability. Moreover, the Cl^−^ ions are stored by neutralizing the changes in net charge that are created by the redox reaction of [Fe(CN)_6_]^3−^ species rather than participating in the redox reaction directly during the desalination/salination process.

The most common and known chloride ion capturing electrode through battery behavior (Ag/AgCl) is silver due to its easy generation and high theoretical capacity (248 mAh g^−1^).^[^ ^20^ ^]^ Chen et al. reported AgCl‐Na_0.44_MnO_2_ electrodes for chloride and sodium capture/release; their salt adsorption capacity was up to 57 mg_NaCl_ g^−1^ for 100 cycles with charge efficiencies of 95.6% (adsorption) and 97.9% (desorption).^[^ ^21^ ^]^ Furthermore, AgCl has also been entangled with Na_3_V_2_(PO_4_)_3_@C wires; its desalination capacity of 98 mg_NaCl_ g^−1^ was achieved at a specific current of 100 mA g^−1^ for more than 50 cycles.^[^ ^22^ ^]^ Instead of implementing Ag or AgCl to other materials, it is also possible to directly use the Ag/AgCl redox couple in a two‐channel setup by use of one cation‐exchange membrane.^[^ ^23^ ^]^ To operate at a low voltage, individual silver and silver chloride electrodes were investigated.^[^ ^24^ ^]^ Such a combination enables operation at a very low cell voltage of only 0.2 V; notably, the above electrodes obtain desalination capacities of 85 and 115 mg_NaCl_ g^−1^ while consuming low amounts of energy. However, during the desalination process, the formation of AgCl at the surface impedes the further diffusion of Cl^−^ and, per its nonconductive nature, impedes electron transport, too. Thus, an ion conductor network that is constructed by coupling Ag with reduced graphene oxide (Ag@rGO) provides high ion mobility and enhanced structural stability due to the 2D sheet structure of the electrode and the flexibility of rGO.^[^ ^25^ ^]^

With regard to Cl^−^‐capture materials, Ag exhibits several superiorities: high desalination performance, fast faradaic reaction, potential stability, corrosion resistance, and bactericidal properties.^[^ ^20^, ^26^ ^]^ However, the relatively high price of silver, the significant particle coarsening of silver during recycling use and the poor electronic conductivity of the formed AgCl limit the practical utilization of Ag/AgCl electrodes.

The development of pseudocapacitive‐behavior electrodes is restricted by a low desalination capacity, although their 2D layer structure and excellent electrical conductivity facilitate ion diffusion and transfer. While a high salt adsorption capacity is achieved, the rate capability of battery‐behavior materials is limited due to unfavorable ion diffusion and the poor conductivity of the formed oxidation substance after the redox reaction at the surface and the significant coarsening of particles during cycling. It is essential to obtain electrodes with a simultaneous fast rate capability and high desalination capacity, which would immensely reduce energy consumption and operating costs. Considering the favorable ion diffusion rate of pseudocapacitive‐behavior electrodes and the high salt adsorption capacity of battery‐behavior materials, coupling the two together can effectively offset their shortcomings; thus, an electrode with a fast rate capability and high desalination capacity is simultaneously achieved.

## 2 Results and Discussion

**Figure 1:**
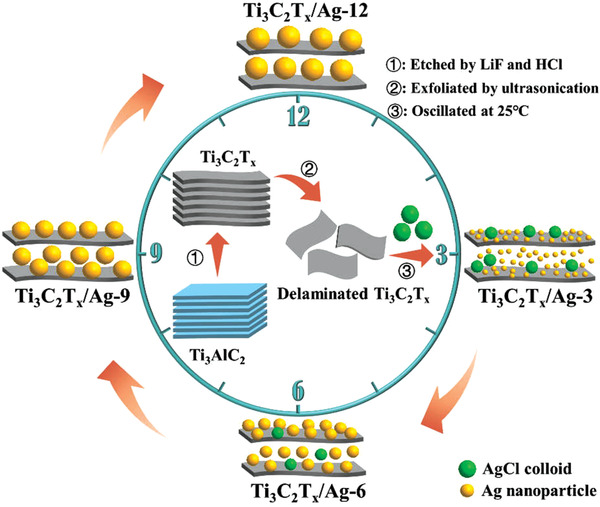
Schematic preparation of Ti_3_C_2_T*_x_*/Ag samples.

In this work, we synthesized Ti_3_C_2_T*_x_*/Ag composites via a facile oxidation‐reduction method at room temperature (**Figure** **1**) and first used it as an anode for chloride ion capture in electrochemical desalination. The AgCl colloid is reduced to Ag nanoparticles by the delaminated Ti_3_C_2_T*_x_*, which is functionalized as the reductant as well as being the active component. The sizes and loading contents of Ag nanoparticles are investigated by selecting different reaction times (3, 6, 9, and 12 h).

**Figure 2:**
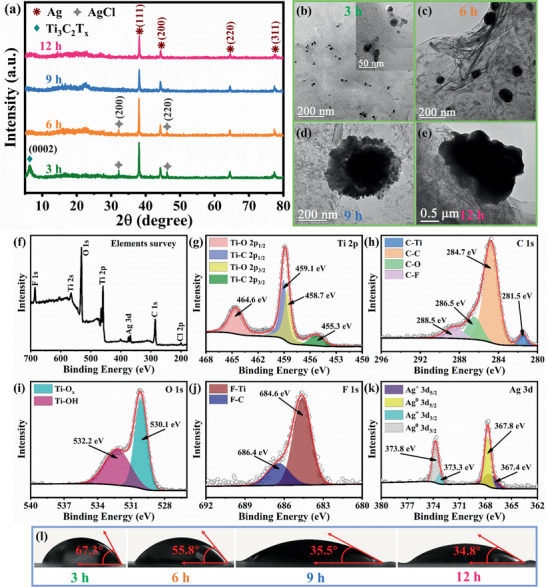
a) X‐ray diffraction patterns of Ti_3_C_2_T*_x_*/Ag prepared with different reaction times and b–e) Transmission electron micrographs of Ti_3_C_2_T*_x_*/Ag with reaction times of 3, 6, 9, and 12 h. X‐ray photoelectron emission spectra of Ti_3_C_2_T*_x_*/Ag‐3: f) survey spectrum, g) Ti 2p, h) C 1s, i) O 1s, j) F 1s, and k) Ag 3d. l) Optical micrographs of the water contact angles on the surface of Ti_3_C_2_T*_x_*/Ag corresponding to reaction times of 3, 6, 9, and 12 h.

**Figure** **2**a shows the X‐ray diffractograms of Ti_3_C_2_T*_x_*/Ag‐3, Ti_3_C_2_T*_x_*/Ag‐6, Ti_3_C_2_T*_x_*/Ag‐9, and Ti_3_C_2_T*_x_*/Ag‐12. All the samples display clear diffraction peaks, corresponding to face‐centered cubic silver. Two other peaks exist at 32.2° 2*θ* and 46.3° 2*θ* in Ti_3_C_2_T*_x_*/Ag‐3 and Ti_3_C_2_T*_x_*/Ag‐6, which belong to diffraction peaks of AgCl. It should be noted that only Ti_3_C_2_T*_x_*/Ag‐3 sample shows the obvious (0002) diffraction peak of Ti_3_C_2_T*_x_*‐MXene derives from the partial stacking of Ti_3_C_2_T*_x_* nanosheets, which relates to the more disordered stacking order with increased reaction time due to the more oxidation of Ti_3_C_2_T*_x_*‐MXene.^[^ ^27^, ^28^ ^]^ The above result indicates that more than 6 h are required to transform AgCl to Ag nanoparticles. The free‐standing film prepared by vacuum filtration exhibits a circle with a diameter of ≈3.8 cm and the thickness of film is ≈5 µm, which can maintain its flexible structure (Figure S1, Supporting Information). The morphology exhibits a stacked, layered structure, of which the surface has wrinkles.

The microstructure of Ti_3_C_2_T*_x_*/Ag was investigated by transmission electron microscopy (TEM). Transmission electron micrographs reveal that after shaking for 3 h, some Ag nanoparticles have grown on the surface of Ti_3_C_2_T*_x_*, while some are situated on the edge of the surface, similar to a bridge linking two exfoliated Ti_3_C_2_T*_x_* particles (Figure 2b). These “bridges” can shorten the transfer distance of the electron from one Ti_3_C_2_T*_x_* to another Ti_3_C_2_T*_x_*. The Ag nanoparticles are spherical with sizes ranging from 20 to 30 nm. As the shaking time increases, Ag particles continually grow (Figure 2c–e), and the size of Ti_3_C_2_T*_x_*/Ag‐12 reaches several hundred nanometers, substantially weakening the contact area with the electrolyte. Accordingly, the size of the Ag nanoparticles increase, and the AgCl colloid disappears gradually as the reaction time is prolonged, which is verified by the X‐ray diffraction (XRD) and TEM images. In addition, less oxidation for Ti_3_C_2_T*_x_*/Ag‐3 did not destroy the ordered stacking interlayer structure (Figure 2a–e), which might be favorable for ion diffusion and intercalation.

The chemical composition and elemental states on the surface of the samples are investigated by X‐ray photoelectron spectroscopy (XPS). As illustrated in Figure 2f, the Ti, C, O, and F are related to Ti_3_C_2_T*_x_*, Ag, and Cl, which are present in the XPS survey spectra of Ti_3_C_2_T*_x_*/Ag‐3. High‐resolution XPS spectra of Ti 2p (Figure 2g) reveal the existence of Ti—C 2p_1/2_, 2p_3/2_, and Ti—O 2p_1/2_, 2p_3/2_ located at 459.1, 455.3, 464.6, and 458.7 eV, respectively. The C 1s spectrum (Figure 2h) can be deconvoluted into four peaks (281.5, 284.7, 286.5, and 288.5 eV), corresponding to C—Ti, C—C, C—O, and C—F, respectively. Figure 2i displays the high‐resolution O 1s spectra that can be divided into two Ti—O*_x_* (530.1 eV) and Ti—OH (532.2 eV). As shown in Figure 2j, the peaks located at 684.6 and 686.4 eV can be assigned to F—Ti and F—C bonds, respectively. The Ag 3d spectra of Ti_3_C_2_T*_x_*/Ag‐3 (Figure 2k) contains two peaks, namely, Ag 3d_5/2_ and Ag 3d_3/2_; each peak can be divided into two peaks at 367.4 and 367.8 eV as well as 373.3 and 373.8 eV, respectively. The peaks at 367.8 and 373.8 eV are attributed to metallic Ag, while the peaks at 367.4 and 373.3 eV belong to Ag^+^ of AgCl, indicating the successful incorporation of Ag nanoparticles that are generated by reducing AgCl on Ti_3_C_2_T*_x_*. This result agrees with the XRD analysis. To evaluate the role of AgCl, the high‐resolution Ag 3d spectra and energy‐dispersive X‐ray spectroscopy (EDX) measurements of Ti_3_C_2_T*_x_*/Ag electrodes were conducted. It was indicated that the AgCl only occurred in Ti_3_C_2_T*_x_*/Ag‐3 and Ti_3_C_2_T*_x_*/Ag‐6 hybrids due to inadequate reaction time (Figure 2a; and Figure S2, Supporting Information). The contents of Ag and AgCl in Ti_3_C_2_T*_x_*/Ag electrodes have been calculated based on EDX measurement (Table S1, Supporting Information). The Ag content is incremental, and the AgCl content is decreased in Ti_3_C_2_T*_x_*/Ag hybrid with increasing the reaction time. When the reaction time exceeded 6 h, the AgCl disappeared. The weight percent of total silver of all samples are almost the same if all AgCl is converted to silver. Residual AgCl would release Cl^−^ to generate Ag during the electrode regeneration due to the electrochemical reversibility. To avoid the effect of residual AgCl, an inverse voltage was applied to wash the Ti_3_C_2_T*_x_*/Ag‐3 and Ti_3_C_2_T*_x_*/Ag‐6 electrode before starting desalination measurements. Thereby, residual AgCl is mostly converted to Ag, which is verified by EDX characterization (Table S2, Supporting Information). Thus, the Ti_3_C_2_T*_x_*/Ag hybrids with different reaction time have almost identical components (Ag and Ti_3_C_2_T*_x_*, only tiny AgCl for Ti_3_C_2_T*_x_*/Ag‐3 and Ti_3_C_2_T*_x_*/Ag‐6) and similar Ag° content (17.21–21.01 wt%) before desalination process. The analysis of XPS spectra demonstrates that the surface of Ti_3_C_2_T*_x_* has abundant —F and —OH groups and that the Ag nanoparticles are successfully decorated onto Ti_3_C_2_T*_x_*.

A good wetting ability has a positive effect on desalination performance.^[^ ^29^ ^]^ Thus, water contact angles were measured to determine the hydrophilicity of the as‐prepared samples (Figure 2l). The water contact angles of Ti_3_C_2_T*_x_*/Ag with reaction times of 3, 6, 9, and 12 h are 67.3°, 55.8°, 35.5°, and 34.8°, respectively. This finding indicates that all four samples are hydrophilic and that the wetting ability improves as the reaction time increases. This is because the hydrophilicity of the samples is enhanced by the increasing content of silver.

**Figure 3:**
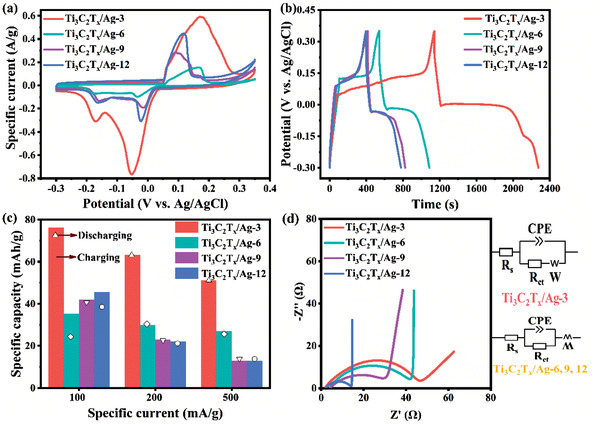
a) Cyclic voltammograms at a scan rate of 0.5 mV s^−1^. b) Galvanostatic charge/discharge with potential limitations profiles. c) Charging and discharging capacities at various specific currents, and d) Nyquist plots from electrochemical impedance spectroscopy measurements of Ti_3_C_2_T*_x_*/Ag‐3, Ti_3_C_2_T*_x_*/Ag‐6, Ti_3_C_2_T*_x_*/Ag‐9, and Ti_3_C_2_T*_x_*/Ag‐12.

**Figure** **3**a shows the cyclic voltammograms of Ti_3_C_2_T*_x_*/Ag. Distinct oxidation and reduction peaks, with similar peak positions as the previous work, are all observed after testing the four samples. The oxidization peaks of Ti_3_C_2_T*_x_*/Ag‐3, Ti_3_C_2_T*_x_*/Ag‐6, Ti_3_C_2_T*_x_*/Ag‐9, and Ti_3_C_2_T*_x_*/Ag‐12 are detected at ≈0.176, 0.169, 0.099, and 0.118 V versus Ag/AgCl, respectively, which is evidence for the formation of AgCl. During the reduction process, the reduction peaks of Ti_3_C_2_T*_x_*/Ag‐3, Ti_3_C_2_T*_x_*/Ag‐6, Ti_3_C_2_T*_x_*/Ag‐9, and Ti_3_C_2_T*_x_*/Ag‐12 are located at −0.052, −0.035, −0.014, and −0.023 V versus Ag/AgCl, respectively, indicating the reversible reaction of AgCl back to Ag. These reduction potentials are far less than those of the recently reported chloride‐storage Bi electrode (−1.27 V vs Ag/AgCl).^[^ ^19^ ^]^ It is demonstrated that better redox kinetics occur at the interface of Ti_3_C_2_T*_x_*/Ag electrodes due to the small size of Ag nanoparticles, while the reduction kinetics indicate that the electrochemical reduction reaction of BiOCl to Bi is sluggish. Compared with individual silver and silver‐chloride electrodes (redox peaks at ≈±0.4 V vs Ag/AgCl),^[^ ^24a^ ^]^ the Ti_3_C_2_T*_x_*/Ag electrodes show lower redox potential, establishing that the introduction of Ti_3_C_2_T*_x_* accelerates the diffusion of ions enabling more electroactive reaction sites.^[^ ^30^ ^]^ Other small peaks may be ascribed to side reactions, such as the reduction peak of the oxygen reduction reaction.^[^ ^31^ ^]^ The peak intensities of the four samples are present in the order of Ti_3_C_2_T*_x_*/Ag‐3, Ti_3_C_2_T*_x_*/Ag‐12, Ti_3_C_2_T*_x_*/Ag‐9, and Ti_3_C_2_T*_x_*/Ag‐6, which is likely related to the efficiency of electron transport and the capacity of the material. Ti_3_C_2_T*_x_*/Ag‐3 delivers a much higher current than Ti_3_C_2_T*_x_*/Ag‐6, Ti_3_C_2_T*_x_*/Ag‐9, and Ti_3_C_2_T*_x_*/Ag‐12, indicating the highest capacity of Ti_3_C_2_T*_x_*/Ag‐3 among those electrodes.

A more quantitative assessment of the capacity of Ti_3_C_2_T*_x_*/Ag is obtained by galvanostatic charge/discharge with potential limitations measurements (GCPL) in 1 m NaCl solution (Figure 3b). All the profiles show plateaus with similar voltage positions, which further demonstrates that battery behavior exists in the charging/discharging process. Figure 3c compares the capacity of these four samples at various specific currents. The charging capacities of Ti_3_C_2_T*_x_*/Ag‐3, Ti_3_C_2_T*_x_*/Ag‐6, Ti_3_C_2_T*_x_*/Ag‐9, and Ti_3_C_2_T*_x_*/Ag‐12 present regularity at all specific currents except 100 mA g^−1^. This is probably due to the more hydrophilic nature and low charge transfer resistance of Ti_3_C_2_T*_x_*/Ag‐12. Regardless of which specific current is tested, Ti_3_C_2_T*_x_*/Ag‐3 always exhibits the highest capacity among the four materials, and its capacity reaches 76 mAh g^−1^ at 100 mA g^−1^, 1.7 times that of Ti_3_C_2_T*_x_*/Ag‐12 (the second most among the four materials). This is because small silver nanoparticles generally have high reactivity and more contact areas with the electrolyte. The charging and discharging capacities of Ti_3_C_2_T*_x_*/Ag‐3 are nearly the same, which indicates high electrochemical reversibility.

To further investigate the electrochemical performance of Ti_3_C_2_T*_x_*/Ag, electrochemical impedance measurements were conducted (Figure 3d). In the Nyquist plots, the intercept of the semicircle on the real axis represents the equivalent series resistance (*R*_s_), and the diameter of the quasisemicircle signifies the charge transfer resistance (*R*_ct_) of the materials. *R*_s_ responds to the sets of electrolyte and interfacial resistance among the electrode, current collector, and electrolyte. Under the same measurement conditions, the *R*_s_ values of the four samples are almost the same. The *R*_ct_ values of Ti_3_C_2_T*_x_*/Ag‐3, Ti_3_C_2_T*_x_*/Ag‐6, Ti_3_C_2_T*_x_*/Ag‐9, and Ti_3_C_2_T*_x_*/Ag‐12 are 45.5, 39.9, 20.8, and 4.1 Ω, respectively, which is affected by the surface wettability and the Ag content.

**Figure 4:**
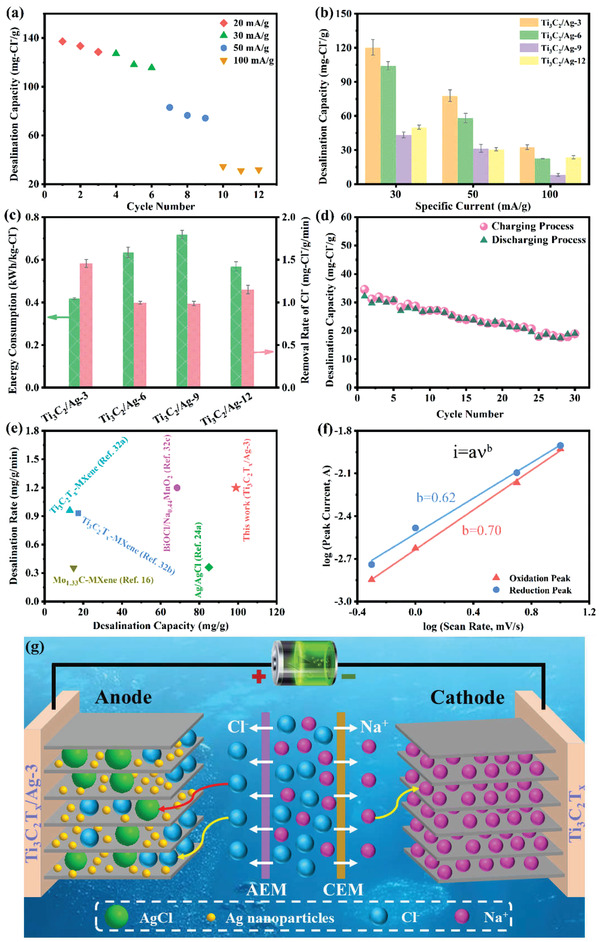
a) Desalination capacity of Ti_3_C_2_T*_x_*/Ag‐3 at specific currents of 20, 30, 50, and 100 mA g^−1^. b) Comparison of the desalination capacity of Ti_3_C_2_T*_x_*/Ag‐3, Ti_3_C_2_T*_x_*/Ag‐6, Ti_3_C_2_T*_x_*/Ag‐9, and Ti_3_C_2_T*_x_*/Ag‐12 at various specific currents. c) Contrast of energy consumption and chloride ion removal rates of the four kinds of materials at a specific current of 50 mA g^−1^. d) Cycling performance of Ti_3_C_2_T*_x_*/Ag‐3 at a specific current of 100 mA g^−1^. e) Comparison of the desalination capacity and rate among the different electrodes. f) Power‐law relationship between peak current and scan rates for Ti_3_C_2_T*_x_*/Ag‐3. g) Desalination mechanism of the Ti_3_C_2_T*_x_*/Ag electrode for electrochemical desalination.

The desalination performance of Ti_3_C_2_T*_x_*/Ag is studied via a dual‐ion device in a 10 × 10^−3^ m NaCl solution. Figure S3 (Supporting Information) shows the changes in the potential and corresponding conductivity of Ti_3_C_2_T*_x_*/Ag‐3 during the charging/discharging process. When a positive current is applied, the voltage increases and the conductivity decreases, corresponding to the capture of Cl^−^ in the anode and Na^+^ in the cathode. Under the condition of a negative current, the conductivity increases due to the release of Cl^−^ and Na^+^ from the electrodes. According to Equation (S1) (Supporting Information), the Cl^−^‐removal capacity of Ti_3_C_2_T*_x_*/Ag‐3 is calculated at various values for the specific currents. As seen from **Figure** **4**a, along with the increase in specific current, the Cl^−^‐removal capacity decreases due to the growing polarization of the electrode.^[^ ^21^ ^]^ At a specific current of 20 mA g^−1^, Ti_3_C_2_T*_x_*/Ag‐3 exhibits a high capacity of 135 mg Cl^−^/(g‐Ti_3_C_2_T*_x_*/Ag‐3) (=110 mg_NaCl_ g^−1^).

Figure 4b compares the Cl^−^‐removal capacity of the four samples at various specific currents. Consistent with the results of the GCPL, Ti_3_C_2_T*_x_*/Ag‐3 always displays the highest Cl^−^ removal capacity, with a value of 120 mg Cl^−^/(g‐Ti_3_C_2_T*_x_*/Ag‐3) (=99 mg_NaCl_ g^−1^) at 30 mA g^−1^, ≈2.8 times that of the lowest one (43 mg Cl^−^/(g‐Ti_3_C_2_T*_x_*/Ag‐9)). In addition to Cl^−^‐removal capacity, Cl^−^‐removal rate and energy consumption are also important factors to evaluate whether the material is promising.^[^ ^12b^ ^]^ As shown in Figure 4c, the energy consumption of Ti_3_C_2_T*_x_*/Ag‐3 is 0.42 kWh kg^−1^ Cl^−^ at 50 mA g^−1^, which is much lower than that of other samples. The Cl^−^‐removal rate is 1.5 mg Cl^−^/(g‐Ti_3_C_2_T*_x_*/Ag‐3)/min, which is attributed to its open, 3D structure which is formed by the growth of silver on the sheet of Ti_3_C_2_T*_x_*. Briefly, the Ti_3_C_2_T*_x_*/Ag‐3 electrode exhibits the highest desalination capacity, fastest Cl^−^‐removal rate, and lowest energy consumption compared with other three samples, originating from the smallest size, the most uniform distribution of silver nanoparticles, and ordered stacking structure of Ti_3_C_2_T*_x_* over Ti_3_C_2_T*_x_*/Ag‐3 hybrid material. The stability of Ti_3_C_2_T*_x_*/Ag‐3 was tested for 30 cycles at 100 mA g^−1^ (Figure 4d). It reveals a small decline of capacity and stable desalination capacities of charging and discharging process during 30 cycles, manifesting excellent cycle‐to‐cycle durability of Ti_3_C_2_T*_x_*/Ag‐3.

The relationship between desalination capacity and the rate is illustrated in the Kim–Yoon–Plot in Figure 4e.^[^ ^16^, ^24^, ^32^ ^]^ Even though it is not suitable to compare diverse systems due to the differences in operational conditions and initial concentration of NaCl, it is apparent that this work exhibits much higher desalination capacity than individual MXene (e.g., Ti_3_C_2_T*_x_*, Mo_1.33_CT*_x_*) materials. Moreover, compared with silver and silver‐chloride electrodes, the as‐prepared Cl^−^‐capture electrode displays a faster rate capability as well as a higher desalination capacity. Accordingly, the excellent desalination performance originates from the synergistic effect between the battery (Ag) and pseudocapacitive behaviors (Ti_3_C_2_T*_x_*), which enhances the desalination capacity and rate, respectively.

To further investigate the electrode process, the power‐law relation (*i* = a*v* ^b^, *i* represents the current, *v* denotes the scan rate) was obtained (Figure 4f). The calculated *b* values of the oxidation and reduction peaks over Ti_3_C_2_T*_x_*/Ag‐3 are 0.70 and 0.62, respectively. A *b*‐value of 0.5 would align with perfect adherence to a diffusion time‐law, and a *b*‐value of 1.0 is found for the ideal case of electrical double‐layer formation (i.e., not limited by diffusion).^[^ ^33^ ^]^ The *b*‐value indicates that Ti_3_C_2_T*_x_*/Ag‐3 simultaneously exhibits battery and pseudocapacitive behaviors, thus providing superior desalination capacity and rate capability.

The desalination process of Ti_3_C_2_T*_x_*/Ag paired with Ti_3_C_2_T*_x_* and equipped with a pair of ion‐exchange membranes is illustrated in Figure 4g. Ti_3_C_2_T*_x_*/Ag and Ti_3_C_2_T*_x_* serve as anode and cathode electrodes, respectively. The desalination process can be described as follows: i) the Na^+^ ions pass through the cation exchange membrane and Cl^−^ ions pass through the anion exchange membrane; ii) the Na^+^ ions diffuse from the surface of Ti_3_C_2_T*_x_* and are intercalated into the interlayer of Ti_3_C_2_T*_x_*; and iii) the Cl^−^ ions diffuse from the surface of Ti_3_C_2_T*_x_*/Ag, with some Cl^−^ ions diffusing into the Ag lattice to react with Ag, thus generating AgCl, while others are reserved by intercalation in the interlayer of Ti_3_C_2_T*_x_*. It is indicated that the Ag nanoparticles are formed and uniformly distributed onto Ti_3_C_2_T*_x_* by the XRD, TEM, and XPS analysis results. The as‐prepared composites are hydrophilic, as verified by the water contact angle tests, which has a positive effect on the electrochemical and desalination performance. Based on the electrochemical measurements, it is demonstrated that the Ti_3_C_2_T*_x_*/Ag samples simultaneously possess pseudocapacitive and battery behaviors and a low charge transfer resistance. Among Ti_3_C_2_T*_x_*/Ag with different reaction times, Ti_3_C_2_T*_x_*/Ag‐3 manifests a Cl^−^‐removal rate of 1.5 mg Cl^−^ g^−1^ min^−1^, a Cl^−^‐removal capacity of 78 mg Cl^−^ g^−1^, the energy consumption of 0.42 kWh kg^−1^ Cl^−^ at 50 mA g^−1^ and an excellent cyclability. As a result, the fast rate capability, high desalination capacity, good cyclability, and low energy consumption of Ti_3_C_2_T*_x_*/Ag are exhibited. The excellent desalination performance is attributed to the following factors: i) the 2D layer of Ti_3_C_2_T*_x_* facilitates ion diffusion; ii) the 3D electron‐transfer structure, which is constructed by Ti_3_C_2_T*_x_* and Ag, can offset the low electrical conductivity of AgCl and accelerate electron transmission between the sheets of Ti_3_C_2_T*_x_*; and iii) the above structures are responsible for the synergistic effect between the battery and pseudocapacitive behaviors over the Ti_3_C_2_T*_x_*/Ag hybrid with the capture of chloride ions due to both an intercalation mechanism and conversion reactions.

## 3 Conclusion

In summary, capacitive deionization is promising desalination technology due to its higher energy efficiency and easier generation compared with traditional desalination technology. However, the application of capacitive deionization is limited due to its low desalination capacity, rate, and stability, thus leading to high cost. Herein, Ti_3_C_2_T*_x_*/Ag electrodes were synthesized via a facile oxidation‐reduction method and then first used as an anode for chloride‐ion capture in electrochemical desalination. All Ti_3_C_2_T*_x_*/Ag samples are hydrophilic and have a low charge transfer resistance. The Ti_3_C_2_T*_x_*/Ag‐3 electrode exhibits fast rate capability (1.5 mg Cl^−^ g^−1^ min^−1^ at 50 mA g^−1^), high desalination capacity (135 mg Cl^−^ g^−1^ at 20 mA g^−1^ in 10 × 10^−3^ m NaCl solution), good cyclability, and low energy consumption (0.42 kWh kg^−1^ Cl^−^). Accordingly, we propose a mechanism in which the desalination performance of Ti_3_C_2_T*_x_*/Ag‐3 can be ascribed to the synergistic effect between the battery (Ag nanoparticles) and pseudocapacitive behaviors (layer‐structure Ti_3_C_2_T*_x_*), which can be used to design electrodes with high desalination capacity and fast rate capability for CDI applications. MXene serves a dual role as an intercalation electrode and facile, electron‐conductive network to capitalize on the Ag/AgCl conversion reaction. The preparation of Ti_3_C_2_T*_x_*/Ag electrodes is facile and feasible, which is favorable for the practical application of electrochemical desalination.

## 4 Experimental Section

### Materials Preparation

Ti_3_C_2_T*_x_* was synthesized by exfoliating commercial Ti_3_AlC_2_ powders similar to previous work. LiF powders (1 g) were dispersed in 20 mL HCl (9 mol L^−1^) by stirring for 20 min. Then, 1 g Ti_3_AlC_2_ (11 Technology Co., Ltd., Jilin Province, China) was added to the solution, and the mixture was stirred for 24 h at 40 °C. The resulting powder was rinsed with deionized water several times until the pH of the centrifugal supernatant was above 6. The obtained wet sediments were then delaminated by ultrasonication under the protection of Ar gas for 1 h. The mixture was centrifuged at 3500 rpm for 10 min, and the supernatant was delaminated Ti_3_C_2_T*_x_*. Ti_3_C_2_T*_x_*/Ag was prepared via a facile modified oxidation‐reduction method.^[^ ^27^ ^]^ In detail, 5 mL of a silver chloride colloid was added to 15 mL (2 mg mL^−1^) of a delaminated Ti_3_C_2_T*_x_* solution (the mass ratio of Ag to Ti_3_C_2_T*_x_* was 1:2). Then, the mixture was oscillated in brown conical flask in a constant‐temperature shaker (TS‐200DC, Shanghai Tiancheng Co., Ltd.) at a constant temperature of 25 °C and a rate of 150 rpm for various hours under dark condition. Next, the products were collected by vacuum filtration with a nylon membrane filter (47 mm diameter, 0.2 µm pore size, Whatman) and washed several times with deionized water. After being dried at ambient temperature, the membrane electrode was obtained by stripping it off from the nylon membrane. According to the difference in shaking time (i.e., 3–12), the samples of Ti_3_C_2_T*_x_*/Ag are labeled Ti_3_C_2_T*_x_*/Ag‐3, Ti_3_C_2_T*_x_*/Ag‐6, Ti_3_C_2_T*_x_*/Ag‐9, and Ti_3_C_2_T*_x_*/Ag‐12.

### Electrochemical and Desalination Measurements

The electrodes used in desalination were prepared by adhering the Ti_3_C_2_T*_x_*/Ag membrane on graphite paper (Beijing Jinglong Carbon Technology Co., Ltd.), while those used in the electrochemical measurements were pure membranes of Ti_3_C_2_T*_x_*/Ag. Cyclic voltammetry, GCD, and electrochemical impedance spectroscopy measurements were carried out in 1 m NaCl solutions using a CHI 600D electrochemical workstation (Shanghai CH Instruments Co.).

Desalination performance was obtained through a batch‐mode method in a dual‐ion device, which consists of a Ti_3_C_2_T*_x_*/Ag electrode, Ti_3_C_2_T*_x_*, an anion exchange membrane and a cation exchange membrane. Constant currents with various densities were applied for the charging and discharging process. An inverse voltage was applied to wash the Ti_3_C_2_T*_x_*/Ag‐3 and Ti_3_C_2_T*_x_*/Ag‐6 electrode before starting desalination measurements. An aqueous NaCl solution with a concentration of 10 × 10^−3^ m and a volume of 40 mL was used as the feed water. The conductivity of the solution was monitored by a conductivity meter (Mettler Toledo S230) to determine the deionization capacity and rate of the charging step.

### Characterization

The morphology of Ti_3_C_2_T*_x_*/Ag was obtained using TEM (JEOL‐2010F). XRD experiments were conducted on specimens using an X‐ray diffractometer (Bruker D8 Advance, Bruker AXS) operating at 40 kV and 40 mA. Nickel‐filtered Cu K*α* radiation (*λ* = 0.154 nm) was used in the incident beam. The water contact angle was measured by an optical contact angle measuring device (POWEREACH JC2000). An XPS analysis was carried out with a Kratos Axis Ultra DLD spectrometer using monochromatic Al K*α* X‐rays at a base pressure of 1 × 10^−7^ Pa. Survey scans were conducted between 1100 and 0 eV and revealed the overall elemental compositions of the sample; additionally, regional scans for specific elements were performed. The peak energies were calibrated by placing the major C 1s peak at 284.6 eV.

## Conflict of Interest

The authors declare no conflict of interest.

## Supporting information

Supporting Information
