# Supplementary material for: Combining Battery‐Type and Pseudocapacitive Charge Storage in Ag/Ti3C2T x MXene Electrode for Capturing Chloride Ions with High Capacitance and Fast Ion Transport
Source: Adv Sci (Weinh). 2020 Aug 27;7(18):2000621. doi: 10.1002/advs.202000621 (PMC7509648; doi:10.1002/advs.202000621)
Supplement: Supplementary file 1 — Supporting Information [file ADVS-7-2000621-s001.pdf]

Copyright WILEY-VCH GmbH, 69469 Weinheim, Germany, 2020.

ADVANCED SCIENCE
Open Access

Supporting Information

for *Adv. Sci.*, DOI: 10.1002/advs.202000621

Combining Battery-Type and Pseudocapacitive Charge Storage in Ag/$Ti_3C_2T_x$ MXene Electrode for Capturing Chloride Ions with High Capacitance and Fast Ion Transport

Mingxing Liang, Lei Wang, Volker Presser, Xiaohu Dai, Fei Yu,* and Jie Ma**

Supporting Information

Combining Battery-type and Pseudocapacitive Charge Storage in $Ag/Ti_3C_2T_x$ MXene Electrode for Capturing Chloride Ions with High Capacitance and Fast Ion Transport

Mingxing Liang[1, 4§], Lei Wang[1, 4, 6§], Volker Presser[5, 6*], Xiaohu Dai[1, 4], Fei Yu[2*], Jie Ma[1, 3, 4*]

[1] State Key Laboratory of Pollution Control and Resource Reuse, College of Environmental Science and Engineering, Tongji University, Shanghai, 200092, P. R. China

[2] College of Marine Ecology and Environment, Shanghai Ocean University, Shanghai 201306, P.R. China

[3] Research Center for Environmental Functional Materials, College of Environmental Science and Engineering, Tongji University, 1239 Siping Road, Shanghai 200092, P.R. China

[4] Shanghai Institute of Pollution Control and Ecological Security, Shanghai, 200092, P.R. China

[5] INM – Leibniz Institute for New Materials, Campus D2 2, 66123 Saarbrücken, Germany

[6] Department of Materials Science and Engineering, Saarland University, Campus D2 2, 66123 Saarbrücken, Germany

---

[§] These authors contributed equally to this work.

[*] Corresponding author

Jie Ma, E-mail: jma@tongji.edu.cn

Fei Yu, E-mail: f-yu@shou.edu.cn

Volker Presser, E-mail: volker.presser@leibniz-inm.de

Calculation method and equations

The Cl^--removal capacity (Cl^--RC), Cl^--removal rate (Cl^--RR) and energy consumption (EC) are calculated using following equations, respectively.

$$\text{Cl}^- \text{-RC (mg-Cl}^- \text{/g-electrode)}=\frac{(C_0 - C_e)\times V \times 35.5\times 1000}{m} \quad (1)$$

$$\text{Cl}^- \text{-RR (mg-Cl}^- \text{/g-electrode/min)} = \frac{\text{Cl}^- \text{-RC}}{\text{t}}\times 60 \quad (2)$$

$$\text{EC (kWh/kg-Cl}^- \text{)} = \frac{i\times \int vdt}{3600\times (C_0 - C_e)\times V \times 35.5} \quad (3)$$

Where the C_0 means the initial concentration of NaCl, mol/L; C_e represents the concentration of NaCl after charging, mol/L; V shows the volume of NaCl, L; m denotes the mass of Cl^- storage electrode, g; t signifies the desalination time, s; i and v expresses the current (A) and potential (V) during desalination process, respectively.

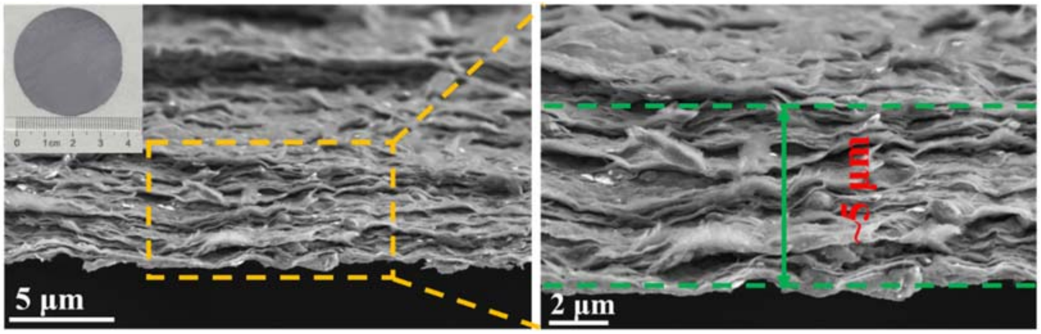

Figure S1. SEM images of $Ti_3C_2T_x$/Ag-3 hybrid (The inset: Digital photograph).

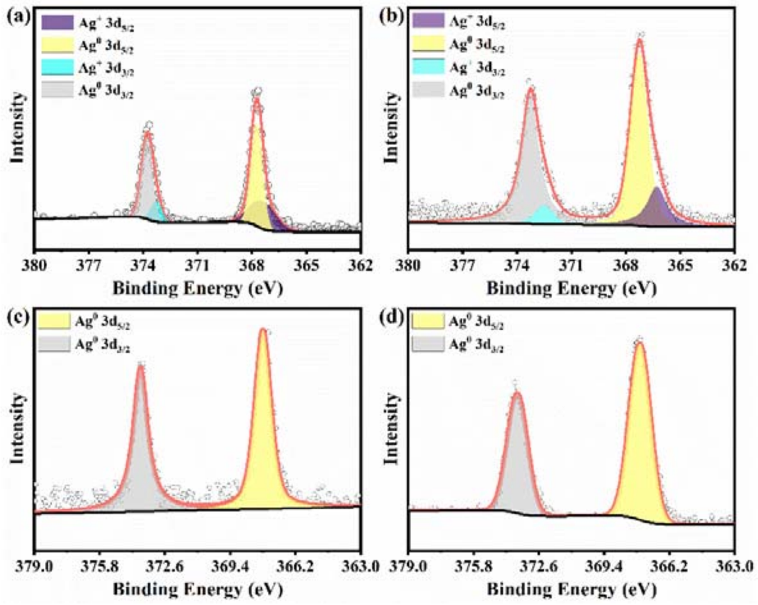

Figure S2. High-resolution Ag 3d XPS spectra of $Ti_3C_2T_x$/Ag hybrid: a) $Ti_3C_2T_x$/Ag-3, b) $Ti_3C_2T_x$/Ag-6, c) $Ti_3C_2T_x$/Ag-9 and d) $Ti_3C_2T_x$/Ag-12.

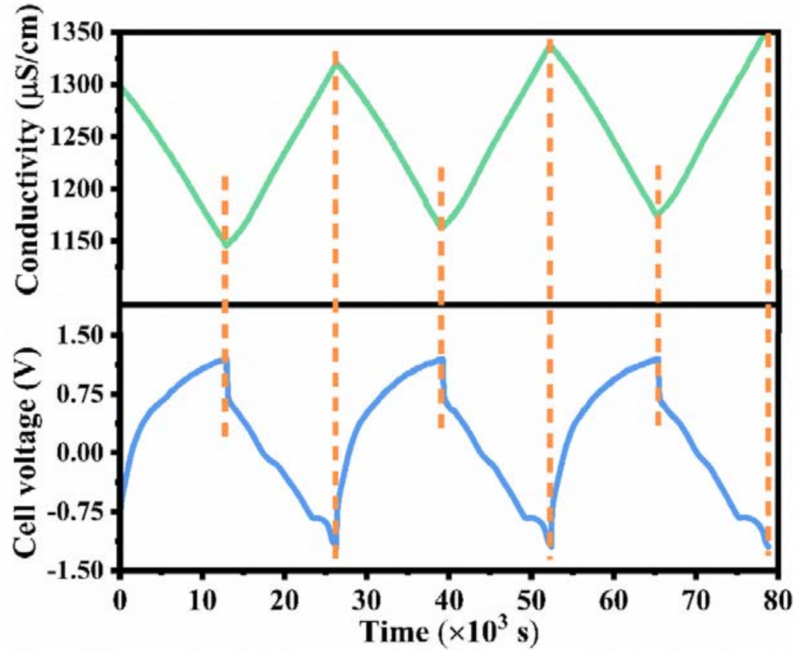

Figure S3. The conductivity (upper) and cell voltage change (lower) during the desalination process of $Ti_3C_2T_x$/Ag-3 at 20 mA/g.

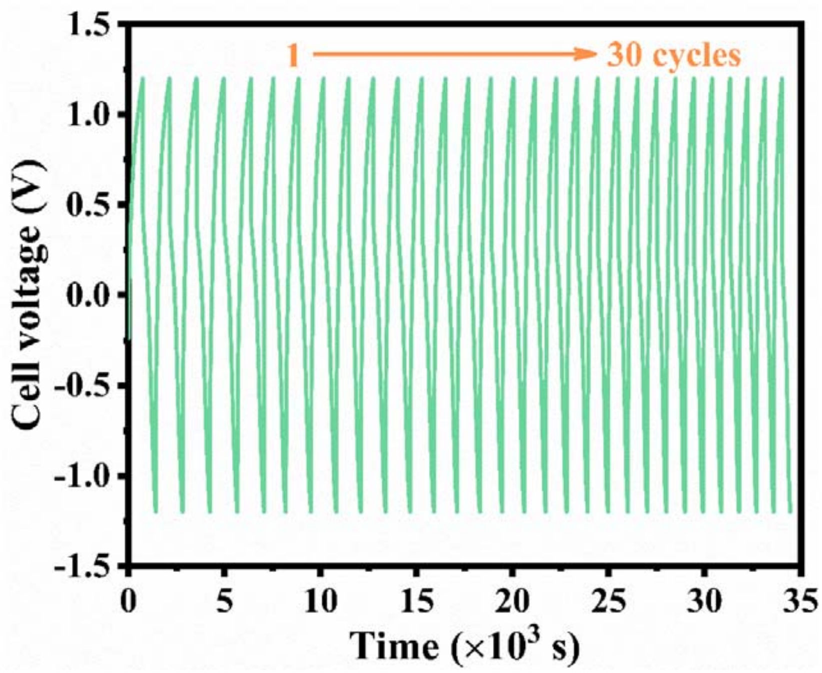

Figure S4. Potential curves of $Ti_3C_2T_x$/Ag-3 electrode at 100 mA/g during 30 desalination cycles.

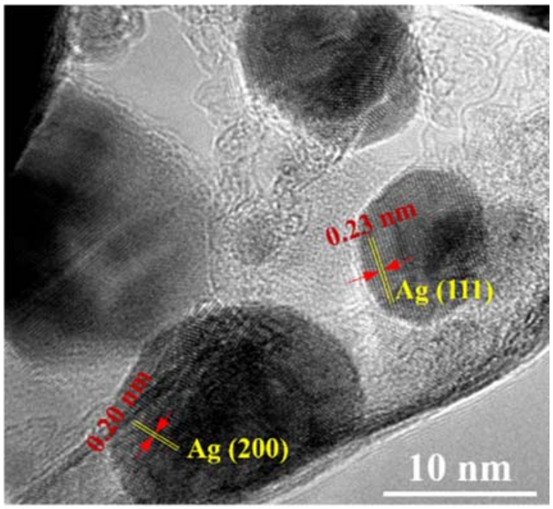

Figure S5. High-resolution TEM image of $Ti_3C_2T_x$/Ag-3 electrode after a long-term cycling.

Table S1. The content of Ag and AgCl species in $Ti_3C_2T_x$/Ag hybrid (EDX measurement).

Electrode materials	Weight percent of Ag^0 (wt%)	Weight percent of AgCl (wt%)	Weight percent of total Ag (wt%)
**$Ti_3C_2T_x$/Ag-3**	7.90	17.68	21.22
**$Ti_3C_2T_x$/Ag-6**	12.52	8.64	19.03
**$Ti_3C_2T_x$/Ag-9**	19.79	-	19.79
**$Ti_3C_2T_x$/Ag-12**	21.01	-	21.01

Table S2. The content of Ag and AgCl species in $Ti_3C_2T_x$/Ag-3 hybrid after different operation (EDX measurement).

**Operation**	**Weight percent of Ag^0 (wt%)**	**Weight percent of AgCl (wt%)**	**Weight percent of total Ag (wt%)**
**Before inverse-voltage washing**	7.90	17.68	21.22
**After inverse-voltage washing**	17.21	6.18	21.86
**After long-term cycling**	19.63	1.90	21.06
